# Challenges and recommendations to improve institutional review boards’ review of community-engaged research proposals: A scoping review

**DOI:** 10.1017/cts.2023.516

**Published:** 2023-03-31

**Authors:** Deborah Onakomaiya, Janet Pan, Timothy Roberts, Holly Tan, Smiti Nadkarni, Marina Godina, Jo Park, Marilyn Fraser, Simona C. Kwon, Antoinette Schoenthaler, Nadia Islam

**Affiliations:** 1 Vilcek Institute of Graduate Biomedical Sciences, NYU Grossman School of Medicine, New York, NY, USA; 2 Department of Population Health, NYU Grossman School of Medicine, New York, NY, USA; 3 University of California, Los Angeles, CA, USA; 4 Human Research Protections, Office of Science and Research, NYU Grossman School of Medicine, New York, NY, USA; 5 National Asian Pacific Center on Aging, Seattle, WA, USA; 6 Arthur Ashe Institute for Urban Health, New York, NY, USA; 7 Institute for Excellence in Health Equity, New York, NY, USA

**Keywords:** Institutional review board, community-engaged research, barriers and facilitators, community participation, community-based participatory research, recommendations

## Abstract

Academic and community investigators conducting community-engaged research (CEnR) are often met with challenges when seeking Institutional Review Board (IRB) approval. This scoping review aims to identify challenges and recommendations for CEnR investigators and community partners working with IRBs. Peer-reviewed articles that reported on CEnR, specified study-related challenges, and lessons learned for working with IRBs and conducted in the United States were included for review. Fifteen studies met the criteria and were extracted for this review. Four challenges identified (1) Community partners not being recognized as research partners (2) Cultural competence, language of consent forms, and literacy level of partners; (3) IRBs apply formulaic approaches to CEnR; & (4) Extensive delays in IRB preparation and approval potentially stifle the relationships with community partners. Recommendations included (1) Training IRBs to understand CEnR principles to streamline and increase the flexibility of the IRB review process; (2) Identifying influential community stakeholders who can provide support for the study; and (3) Disseminating human subjects research training that is accessible to all community investigator to satisfy IRB concerns. Findings from our study suggest that IRBs can benefit from more training in CEnR requirements and methodologies

## Introduction

Community-engaged research (CEnR) is a widely adopted approach when addressing community-level health concerns, as it aims to partner with communities, build leadership, prevent exploitative research practices on vulnerable populations, and enhance the capacity for community participation in research [1]. CEnR is defined as “the process of working collaboratively with and through groups of people affiliated by geographic proximity, special interest, or similar situations to address issues affecting the well-being of those people” (CDC, Principles of Community Engagement, 2005) [1]. Understanding the social, environmental, and cultural context in which an individual lives is crucial for addressing how health issues are perceived and can be targeted. Documented examples of exploitative research practices on racial/ethnic and underserved communities have led to distrust of academic research [2,3]. CEnR is a framework that attempts to undo the distrust of academic research through collaboration and engagement of community members as participants in research, rather than the subjects of research. The principles of CEnR are founded on establishing a mutual relationship between researchers and the community and recognizing the wealth of knowledge that community members possess. CEnR requires the establishment of trust, collaboration, and negotiation in order to identify and address health issues that affect the community.

This approach encompasses a spectrum of strategies and research methodologies in which researchers collaborate with community partners to identify health disparities that affect the community as well as the strengths, preferences, and priorities of community partners [4,5]. As the level of community engagement and involvement increases, so does the ability of communities to be equal representatives in the research process. For example, community-based participatory research (CBPR) is one such orientation that sits on one end of the spectrum of CEnR with the highest level of community involvement, in which there exists a strong bidirectional relationship between the community and researchers with the decision-making driven at the community level [1]. This research orientation seeks to build trust and relationships with community partners to better meet the needs of the community and address health priorities and health disparities [6,7]. CBPR has been a more widely adopted approach for improving public health outcomes [8,9]. Studies have used the CBPR approach to identify health issues and address health outcomes such as chronic diseases, environmental health concerns, workplace health, and infectious disease prevention, and control [10,11].

Prior to beginning any investigative studies with human participants, research must be approved by a research ethics review committee, typically referred to as an Institutional Review Board (IRB) in the USA. IRBs are independent research ethics committees charged with the responsibility of protecting the rights, welfare, and well-being of human subjects involved in any research that involves human participants. There are independent and academic center-affiliated IRBs. Under the Office for Human Research Protections, an IRB is meant to uphold federal standards to prevent the exploitation of human participants in research. Upon review of any form of research involving human participants, the IRB has the authority to approve, modify, or disapprove the research proposal.

Though many goals of CEnR align with that of the IRB to protect human research subjects from unethical harm, researchers and community members conducting CEnR are often met with challenges when seeking to obtain research approval. Because CEnR research design greatly differs from traditional biomedical research methodology, IRB review and approval present unique challenges for CEnR researchers. Previous articles have explored and examined the experiences of community-engaged researchers with the IRB process [12]. These studies have noted several challenges in working with IRBs to review and approve CEnR protocols. However, to our knowledge, there has been a lack of literature that examines these challenges, promising practices and lessons learned from CEnR researchers engaging with academic and affiliated IRBs, and recommendations for navigating the review process.

The aim of the scoping review is to identify challenges that researchers and community partners encounter as well as promising practices that researchers and community partners utilize when working with IRBs for study review of CEnR studies conducted in the USA. We hope that the identified challenges and lessons learned can guide recommendations to better facilitate the research approval process.

## Method

Following the PRISMA guidelines for scoping reviews (PRISMA-Scr), a review was conducted for the available studies describing ways in which IRBs engage CEnR [13]. In August 2019, a trained medical librarian (TR) performed searches for studies without language or date restrictions in MEDLINE, PsycInfo (using the Ovid Platform), and CINAHL (using the Ebsco Platform.) The Ovid Medline search strategy is available in the supplementary materials of this article.

Articles were included in the review if the study (1) reported on CEnR; (2) specified study-related challenges, barriers, lessons learned, or guidelines for working with an IRB; (3) published in a peer-reviewed journal; (4) conducted in the USA; and, (5) had the IRB located in the USA and connected to an academic institution.

Systematic reviews and meta-analyses on related topics were excluded from the scoping review to avoid duplicate studies. However, individual studies listed in these excluded systematic reviews and meta-analyses were tagged for further review. CEnR study proposals by institutional, ethical, or community review boards that were not conducted in conjunction with an academic IRB were also excluded from this review. For this review, we sought to identify and document challenges and facilitators when seeking approval for CEnR from an IRB located at an academic institution.

The title and abstract screening were completed by a team of two co-authors (HT, JP) who identified studies that included academic researchers conducting a CEnR study. Additional criteria included articles that specified study challenges, barriers, lessons learned, promising practices, or facilitators for working with an IRB that was affiliated with an academic institution. These co-authors independently screened titles and abstracts based on the inclusion and exclusion criteria to determine inclusion status. Each citation received two votes from the co-authors and conflicts were solved through consensus or with a third co-author (DO). Two of the co-authors (DO, JP) then conducted a full-text review of the included abstracts to ensure that the articles aligned with the established inclusion and exclusion criteria. Those that did not fit the criteria were excluded. Data extraction was completed with the final set of articles. An extraction table template was designed to extract key barriers, facilitators, and recommendations from CEnR studies, study demographics, and study type. This template was designed using *Covidence,* this software ensured parsimony in the extraction process for each article. Reviewers independently extracted data and the software compared information extracted by each reviewer. Differences in extracted information for each article were discussed between reviewers and the information was consolidated and finalized into one data template.

The elements for extraction included the following: study aim and design, participant demographics, recruitment methods, sample size, description of community partnerships, types of CEnR conducted, types of review boards, challenges, lessons learned, and recommendations for engaging with IRBs and review board research outcomes (i.e. research approval or denial). This final set of articles were independently reviewed for data extraction by two authors (DO, JP). These authors convened to review and finalize the extracted data for analysis.

## Results

**Fig. 1.**
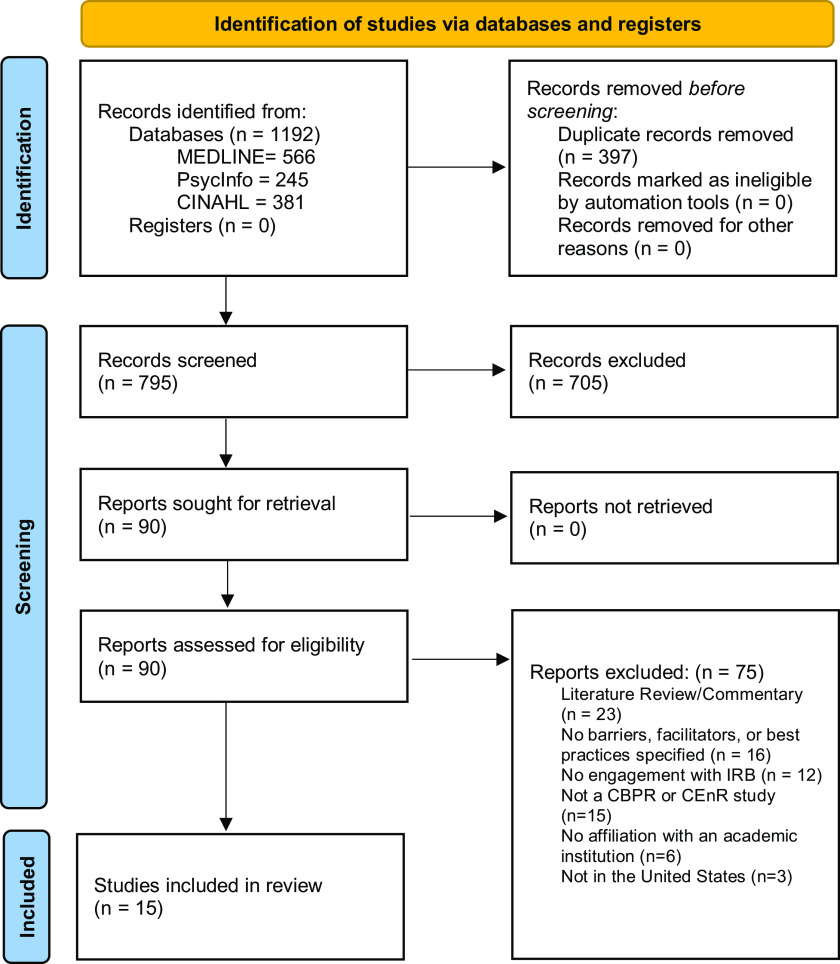
PRISMA 2020 flow diagram for new systematic reviews which included searches of databases and registers only. *From:* Page MJ, McKenzie JE, Bossuyt PM, Boutron I, Hoffmann TC, Mulrow CD, et al. The PRISMA 2020 statement: an updated guideline for reporting systematic reviews. BMJ 2021;372:n71. doi: 10.1136/bmj.n71. For more information, visit: http://www.prisma-statement.org/.

In total, 1192 articles were identified from the database searches. After the removal of duplicates, 795 articles were screened by title and abstract, 705 articles were deemed irrelevant and 90 articles were selected for full-text assessment. Seventy-four articles were excluded using the following exclusion criteria: (1) The article was a literature review, commentary, or letter to the editor (*n* = 23); (2) The article did not specify barriers, facilitators, or recommendations for engaging with IRBs (*n* = 16); (3) The article did not report engagement with IRB (*n* = 12); (4) The article was not a CEnR study (*n* = 15); (5) The IRB was not affiliated with an academic institution (*n* = 6); and (6) The study was not conducted or reviewed in the USA (*n* = 3). Thus, 15 articles met the eligibility criteria and were extracted and included in this review (See Fig. 1).

### Study and Population Characteristics

Most articles were published between 2004 and 2016. Studies were characterized as an evaluation study (*n* = 2), survey questionnaire/report (*n* = 6), qualitative focus group study (*n* = 1), case/expert report (*n* = 4), pre-post (*n* = 1), or mixed-method CBPR collaboration (*n* = 1). When specified, most studies sampled people from racial and ethnic groups (Black, Hispanic/Latinx, and American Indian) [14–20]. Sample sizes ranged from 10 to 900 depending on the study design. While age was not specified in most studies, most participants were adults with some studies engaging older adults 65+ [15–18,21] and teenage/young adults [14,18]. When specified, community partners included public schools, immigration services, youth collaborators, non-profit organizations, health providers, American Indian organizations, and other racial and ethnic community social service organizations [14–18,21–25]. (See Table 1)

**Table 1.** Demographic characteristics of all study populations included in the scoping review

Study	Study type	Sample size	Populations (race/ethnicity)	Community partners
Pirie *et al*., 2013 [18]	Survey report	Not Available	Black and Hispanic/Latinx	Community partners with experience working with the Haitian and Salvadoran communities, academic partners, youth collaborators, cultural coalition groups
Chatterjee *et al*., 2016 [14]	Mixed-method evaluation Study	31	Black and Hispanic/Latinx	Public schools, community health centers, school health centers, teachers, and students
Holkup *et al*., 2004 [15]	Expert report	Not Available	American Indians	American Indian families and elders, social workers, and tribal leaders in the community
Hyatt *et al*., 2009 [24]	Expert report	Not Available	Not Available	Community leaders, community organizations (Immigrant Service Providers Group/Health and Cambridge Health Alliance), and teen educators. All partners had strong connections to the local community.
Johnson *et al*., 2009 [16]	Mixed-method community-based participatory research collaboration	515	Black	Local Somali community organizations, researchers, Immigration Resource Center (a health consultancy and advocacy organization specializing in immigrant and refugee health), public health department, and healthcare providers
Andrews *et al*., 2013 [22]	Program/process evaluation study	Not Available	Not Available	Community-based civil rights organization
Fullerton *et al*., 2015 [21]	Survey questionnaire	101	Not Available	Principal investigators, research assistants, postdocs, students, and community health workers
Paskett *et al*., 2008 [36]	Survey report	Not Available	Not Available	Not Available
Shore *et al*., 2007 [42]	Qualitative research study	10	Not Available	Not Available
Shore *et al*., 2011 [19]	Online qualitative survey	109	African American, Hispanic/Latinx, Mixed, American Indian, White, Pacific Islander	Not Available
Shore *et al*., 2011 [20]	Survey report	109	African American, Hispanic/Latinx, Mixed, No particular racial/ethnic group, American Indian, White, Pacific Islander	Not Available
Silverstein *et al*., 2008 [25]	Survey report	196	Not Available	IRB directors, IRB chairs, and community-based researchers
Brown *et al*., 2010 [23]	Case report	12	Not Available	Environmental health research organization, environmental justice organization
Hueston *et al*., 2006 [43]	Expert report	Not Available	Not Available	Not Available
Malone *et al*., 2006 [17]	Case study	Not Available	Black	Community member focus group volunteers

Additionally, Tables 2 and 3 detail each of the 15 studies included in this scoping review. They provide an overview of each of the study goals and discuss the challenges/barriers and lessons learned implemented in the studies as well as future recommendations for CEnR researchers to implement when they encounter similar challenges.

**Table 2.** Primary research studies documenting challenges and recommendations for engaging with IRBs

Study reference	CEnR	Challenges/barriers	Lessons learned(implemented)	Recommendations (suggested, Not yet implemented)
**Primary research**				
Pirie *et al*., 2013 [18]	The project, Assessing and Controlling Occupational Health Risks for Immigrants in Somerville, Massachusetts involved community partners and an academic partner. Bilingual teen educators were engaged to gather information on self-identified immigrant workers living or employed in the city	1. Informed consent form was too complicated and written at too high of a literacy level for the expected participants 2. IRB process was delayed for approval to start the survey because several corrections were required to be made to the survey flow	1. Supervision of non-native speakers by a bilingual adult leader helped to provide oral consent 2. Human studies certified interpretation was available for non-English speakers 3. Delay in IRB approval led to opportunities to deepen mutual relationships of trust, expand community capacity through education in human participants protection, and support the CITI certification of community partners	Not Available
Chatterjee *et al*., 2016 [14]	Pilot nutrition program launched by community partners and evaluated by investigators at Harvard Pilgrim Health Care Institute (HPHCI) on the impact of a new school food program on the health of their students. School staff and community health centers were involved as stakeholders in the research process. Designed as a community-academic partnership	1. Community partners were not connected with academic IRB, which made it difficult to receive IRB approval as the IRB expressed concerns about the use of community investigators and the ability of academic institutions to ensure human subjects research protections 2. Academic IRBs were mostly familiar with large databases, secondary data analysis projects, and projects involving primary data collection, with little to no experience with CBPR projects 3. The IRB expressed concerns about several of the CBPR components of the study, including supervision for human subjects research protections at a site remote from the academic institution 4. Delays in collaborating partner research approval, which postponed the approval of the project from other collaborating partners and the start of the project 5. Voluminous consent text and language required by the IRB limited recruitment and enrollment of participants into the study. The consent text was not accessible at literacy level and reading levels for parents in the community 6. The institution’s inflexibility with regard to human subjects’ protocols related to CBPR projects led to an imbalance of power by prioritizing the academic institution’s demands over the needs of the community organizations	1. Investigators used training materials that Harvard’s CTSI developed for community investigators in CBPR projects, which covered important topics for all investigators to understand when conducting human subjects research 2. The IRB allowed community and student investigators to participate in certain aspects of the study if they completed training from the institution’s CTSI on human subjects research and CBPR in addition to the HSR training required	1. Disseminating human subjects research training that is accessible for all community investigators, including student investigators, will be an important way to satisfy IRB concerns 2. Institutions such as the Medical University of South Carolina have dedicated paperwork and IRB pathways for researchers seeking review for community-engaged projects. This help to streamline the review process for CBPR projects while ensuring that investigators maintain appropriate human subject safeguards 3. Training IRBs to understand CBPR principles will streamline, reduce delays, and increase the flexibility of the academic IRB review process. 4. Working with IRB offices to develop processes supportive of CBPR and training tools for community investigators can remove many barriers and maintain long-term relationships in communities
Holkup *et al*., 2004 [15]	Used family conference model to resolve problems with American Indian families struggling to care for an elder or resolve problems with a mistreated elder. This model provides a way for families to resolve problems while maintaining self-determination. The research team included American Indian community stakeholders	1. IRB requirements for informed consent resulted in extensive document preparation as they were inflexible to change 2. Interrupting the natural flow of a group interview to gain informed or written consent for a laborious document, when it has been provided implicitly seems inappropriate, especially for action research (also known as community-based research) 3. Difficulty in gaining true informed consent for action research. Because it is the nature of action research to allow the project to evolve as the research progresses, it was not easy to specify explicitly what involvement in the research will mean for the participants 4. External IRBs were less flexible and required all written materials to be physically stamped and all interactions with the community to be scripted	1. Identifying key stakeholders with influence in the community who can provide support for the research study 2. Having a review board that is sensitive to cultural concerns and therefore will allow necessary flexibility to the research project, especially for projects that have a CBPR component which often needs to have sufficient leeway for the project to follow its natural evolutionary course	Not Available
Hyatt *et al*., 2009 [24]	To determine whether an education and training intervention involving both community partners and IRB leadership could resolve issues around gaps in CBPR knowledge and mistrust. This intervention was aimed at identifying and controlling occupational health and safety risks among immigrant workers in Somerville MA. The goal was to give community leaders, particularly in partner organizations, additional experience, and training in survey development, implementation, and analysis, with assistance provided by coinvestigators at Tufts University	1. In working with immigrant workers, human subjects’ protection was a critical issue, but community partners were not knowledgeable about the need for such protections or the role of the IRBs	1. IRB issues were communicated as often and as clearly as possible, with both community partners and their own IRB to minimize last-minute surprises 2. When a study involves human participants and is carried out by collaborators with a wide range of backgrounds, all verbal communications should be followed up with a written memo to both community partners and the IRB3. Successful participation in IRB-certified activities with university and medical partners adds considerable depth and validity to a broad spectrum of grant applications made by community partners. Therefore, encouraging IRB training for community partners engaged in the CBPR process is important	1. It is critical for academic researchers to involve their community partners with the IRB as early in the research process as possible 2. Mistrust of science and research is widespread in the general population, but meeting with an IRB administrator representative can lessen the mystique of IRB oversight for the community partners, elucidate the goals and process of the IRB, and help to establish trust on both sides 3. Face-to-face open discussion of intentions and goals shows all parties that they share the aim of both protecting community members and giving them a voice, as a remarkable amount of social capital for the university and the community partners is created in the face-to-face meetings and subsequent interactions 4. Academy-based CBPR researchers must recognize the need for a transfer of experience and formal training on IRB issues to community members when planning a CBPR project and must include time, expertise, and cost for this in the research proposal 5. Researchers provide examples from their own research experience to reinforce the traditional human participant protection examples found in the literature 6. Communicate about IRB issues as often and as clearly as possible, with both community partners and IRB, to minimize last-minute surprises
Johnson *et al*., 2009 [16]	To examine Somali immigrant women’s experiences with the U.S. healthcare system, exploring how attitudes, perceptions, and cultural values, such as female genital cutting, influences their use of reproductive health care. Incorporating screening health surveys, in-depth semi-structured focus groups, and individual interviews with Somali women and local healthcare providers who care for the Somali community. Community members engaged throughout the research process	1. Distrust with the process of informed consent. Participants were reluctant to sign consent documents, for fear of written consent as a possible means of identification. Participants in the community are often averse to signing official-looking documents and have varying literacy levels 2. The practice of obtaining written consent may be problematic in certain refugee communities, as orally-based cultures may assign a higher value to verbal consent, and the presentation of a multi-page document may be irrelevant, inscrutable, or perceived as intimidating	1. The IRB provided a waiver of written/signed informed consent, wherein documentation of verbal consent obtained through an interpreter was sufficient	1. Conducting studies involving refugee communities may necessitate educating IRBs on cultural nuances that may fall outside the boundaries of western values of individual autonomy, self-determination, and freedom inherent to the concept of informed consent
Andrews *et al*., 2013 [22]	Training program for community and academic partners’ collaborative research (i.e. A community-engaged scholars’ program)	1. IRB had issues with community members listed as co-investigators on IRB applications	1. Having an institutional faculty member with CBPR knowledge and experience to serve as part of the IRB group 2. Providing training to IRB administrators, faculty, and community partners on the IRB process for community-engaged research3. Implement a process to allow community partners access to the electronic IRB application and acknowledgment as a co-principal investigator, as well as instructional guidelines, templates, and algorithms to enhance the IRB navigation process	Not Available

CBPR, community based participatory research; CEnR, community-engaged research; CTSI, Clinical and Translational Science Institute; IRB, Institutional review board

**Table 3.** CEnR investigator/expert reports documenting challenges and recommendations for engaging with IRBs

Study reference	CEnR	Challenges/barriers	Lessons learned(implemented)	Recommendations (suggested, not yet implemented)
**CEnR Investigator/Expert Report**				
Fullerton *et al*., 2015 [21]	A paper-based self-administered anonymous survey was distributed at the end of a plenary session to capture the Centers of Population Health and Health Disparities researchers' experiences with institutional oversight of CEnR and CBPR over the course of the researcher’s career	1. The consent forms required by the IRB were burdensome, too long, technical, or too complex, a concern which may or may not reflect the community-oriented nature of study protocols 2. Difficulties with the IRB not recognizing an ethical concern specific to the community 3. The IRB raising concerns about community involvement 4. IRB requiring substantial changes to the protocol that affected community involvement 5. Limited awareness of majority regulatory mechanisms: only 31.7% heard of Institutional Review Board Authorization Agreements (IAAs) with 17.8% having used them in the CEnR/CBPR context. Only a quarter heard of Individual Investigator Agreements (IIAs), with 11.9% aware of IIAs used in the CEnR/CBPR context	1. Submitting research proposals to a dedicated IRB panel for CBPR (e.g. American Indian nations often have their own IRBs) 2. Most researchers sought out Federal Wide Assurance (FWA) for community partners. An FWA is a statement of principles governing an institution’s approach to the protection of human subjects to permit the extension of human subjects oversight to community partners who are not otherwise affiliated with an academic research institution) 3. Having an IAAs, for CBPR/CEnR research allows entities that have an FWA to use an IRB at another assured institution for the review of their research. This reduces the burden of establishing an independent IRB and helps streamline the review of collaborative research conducted by multiple assured institutions, as would be the case in an academic-community partnership 4. Having an IIA for CBPR/CEnR research allows community collaborators not otherwise affiliated with an assured institution to conduct research under the supervision of a principal investigator from an assured institution	1. Academic institutions with researchers who frequently employ CEnR and/or CBPR approaches in their work should take steps to ensure that their IRBs bring to investigators’ attention all potentially relevant regulatory mechanisms, including those like the IIA which might allow investigators to collaborate with non-assured community partners and partner entities 2. Researchers and academic institutions need to improve capacity-building assistance for research among community partners, which should also extend to research oversight. This should not wait until the IRB application has been submitted, but perhaps should start before funding is secured 3. IRBs require a check-off on initial submission forms as to whether there are community partners without an IRB of their own and providing IRB training that covers these mechanisms 4. IRB training and prompts on submission forms, that are tailored to investigators partnering with community organizations are probably the best way to ensure broader awareness of CBPR and CEnR research
Paskett *et al*., 2008 [36]	To describe the recruitment experiences of projects that actively recruited minority and underserved populations as part of the Centers for Population Health and Health Disparities (CPHHD) initiative and to provide examples of strategies employed to aid in recruiting underserved populations to research studies. The study describes multiple sites engaged in CBPR and recruitment issues	1. Length of time to obtain IRB approval led to delays in beginning recruitment 2. IRBs are overburdened and restricted the number of amendments that can be submitted at a time 3. Need for different additional certificates of confidentiality or consent 4. Difficulties in coordinating IRB approval at multiple institutions5. Issues with using non-university affiliated clinics and clinic staff for recruitment6. Need for HIPAA-related requirements e.g. private spaces for interviewing and privacy guidelines	1. Protocol changes to respond to recruitment difficulties offering extra assurances of confidentiality	Not Available
Shore *et al*., 2007 [42]	To examine the experiences of CBPR researchers with the IRB process and to generate recommendations for research teams and IRB reviewers regarding ethical and effective reviews of CBPR projects	1. Completion of the IRB process can be time-consuming 2. A sense of disconnect occurs when researchers feel that the IRB inadequately understands their research 3. Inappropriate IRB feedback attributable to a lack of expertise in a given substantive area, as well as a rigid review process can potentially create situations that increase the risk to study participants	Not Available	1. A more diverse committee is needed to appropriately anticipate potential risks and benefits of CBPR projects 2. Forming a community-based IRB comprised of community members as well as members of the non-profit community and academia 3. Address assumptions that negatively impact non-traditional research approaches 4. IRB reviewers would benefit from having a basic understanding of CBPR 5. Strategies to facilitate mutual education included training, developing guidelines, serving on the IRB, and inviting IRB reviewers to CBPR events. Guidelines should not be handed down by the IRB, but rather developed by the CBPR field and/or through a collaborative effort between IRBs and CBPR teams 6. After receiving IRB approval, there is a continued need to communicate with the IRB particularly when a project needs to be modified. Work with the IRB up-front to create a system that allows for quicker turnaround time for approval or have the IRB assign a lead contact person to each research project, who would be familiar with the project and therefore better positioned to respond more quickly to the researcher 7. CBPR field could generate guidelines to assist IRB reviewers 8. Inform the IRB by writing the project meets the exemption or minimal risk review criteria. This strategy does not imply misrepresenting the project’s intent but rather speaks to design considerations 9. Simplify the proposal and write to your audience (i.e. the IRB). This may entail excluding references to the partnership agreements and instead focusing on the anticipated IRB concerns. If the IRB is unfamiliar with CBPR, mentioning partnership issues may cause confusion 10. Provide as much information up-front as possible, particularly for projects within an emergent design. Even if the specifics of the design cannot be mapped out, the research team should at least try to outline the general domains of the types of questions that the project will examine.
Shore *et al*., 2011 [19]	To systematically describe community-based processes for ethics review of research in the United States	1. Recruitment and support of engaged reviewers 2. Coordination with external entities, and infrastructure to support the review process 3. Time required to co-ordinate one’s review process with other entities, such as different community groups or other ethics review processes 4. Time required of volunteer reviewers to prepare for and attend the review meetings 5. Staff time is required to coordinate the process, which often is an added responsibility to their already busy schedule 6. Time required to discuss and review protocols, especially when competing agendas or diverse perspectives exist among reviewers 7. Differences in underlying priorities or values e.g. conflict with other IRBs that do not address group harm 8. Reviewers are more familiar with traditional forms of research and often do not understand community involvement	Not Available	1. IRBs would need to increase their understanding of CEnR, strengthen their community composition, and explicitly include community-level ethical considerations, in their policies, processes, and application forms 2. A system involving community-based and institution-based research ethics review may be the ideal process for CEnR review
Shore *et al*., 2011 [20]	To discuss a systematic study in the United States of the community-based review process (CRP) through an online survey of community groups and community-institutional partnerships involved in conducting human subjects research and/or advising on its conduct. Survey report of community-based review processes and their relationships with institution-based research ethics boards	1. A significant amount of time and effort is required for preparing an IRB submission and there are also time delays in working through requirements that conflict with community needs and timeframes 2. Communication problems3. IRBs often lack understanding of CEnR4. Difficulty in resolving conflicts with multiple research ethics boards especially conflicts between IRB and community-based review processes	1. Strengthening communication and coordination between community-based review processes and IRBs may lead to improved understanding of each other’s roles and contexts, stronger working relationships, and ultimately more efficient and thorough reviews of CEnR	1. If the CRP is a community review ethics board (C-REB) and there is an academic partner involved in the study, the study could be reviewed by both the C-REB and the Institutional Review Ethics Board (I-REB) simultaneously or sequentially 2. If the CRP is not a C-REB and there is no academic partner involved in the study, the need for an I-REB review will depend on the specifics of the study 3. A system involving community-based and institution-based research ethics review may be the ideal to strive for, despite the inevitable challenges and complexities involved 4. There is potential for a more thorough and effective ethics review of CEnR if community groups/partnerships with research ethics review processes and IRBs routinely communicated
Silverstein *et al*., 2008 [25]	To describe the range of IRB requirements for, and approaches to community-based research (CBR). Describing and ultimately reducing this variability may also be important to providing consistent benefits to all communities participating in CBPR	1. Majority of IRBs reported that they would serve as a Federal Wide Assurance (FWA) designated review board, and a substantial proportion indicated that they would charge a fee for doing so. Institutional liability was the most common reason for an unwillingness to serve as a designated IRB. 2. High variability in approaches to obtaining IRB review for community organizations engaged in CBR; high level of variability in determining who takes responsibility for IRB review for, non-assured community researchers (i.e. researchers not covered under an FWA) may also have implications for the conduct of health disparities research	Not Available	1. Policies and best practice guidelines for how IRBs consider research involving unaffiliated organizations should come not only from individual institutions but also from regulatory agencies with enough credibility to encourage consistency across institutions 2. Efforts to encourage more uniform practices must not only consider the protection of human subjects, but also address institutions’ perceptions of liability, investigators’ responsibility for oversight, and how relationships between academic and community organizations are codified 3. Within institutions, offices that handle grants and contracts should coordinate their policies with those of administration and the IRB in deciding what types of research an institution can support, and what additional funds if any, need to be added to investigators’ budgets to ensure appropriate IRB review
Brown *et al*., 2010 [23]	To highlight problems that arise when Belmont principles meet the IRB review process, and how those problems undermine rather than support the spirit of the Belmont Report. Using accounts with reports from other CBPR researchers, this article proposes procedures that will allow IRBs and CBPR researchers to work together in a review process that promotes their shared goal of ethical, principled, and beneficial research	1. IRB has become too formulaic and inflexible, often the process can become costly and cause long delays 2. The IRB process was designed around biomedical and behavioral research, making the IRB review process inappropriate to the methods, challenges, and objectives of other approaches to research like CBPR 3. Layperson participation in the research process is often outside the conventional jurisdiction of institutional IRBs. This is due to their capacity to oversee and hold accountable the research work of independent organizations that were not legal entities of the university 4. The CBPR philosophy of openness and the practice of reporting back, challenges IRB assumptions about who controls the flow of data produced in human subjects research, when and whether those data should be made available to members of an affected community, and what the nature and duration of the researcher-subject relationship should be 5. IRBs frequently express concerns that disseminating uncertain data may harm human subjects 6. IRBs express concerns when community-based organizations (CBOs) serve as formal partners in a research initiative due to institutional jurisdiction. IRBs are reluctant to oversee human subjects protection compliance for partner organizations outside the university 7. IRBs may be particularly disturbed when a CBO challenges traditional academic norms by engaging in both research and advocacy, leading IRBs to attempt to influence activities that many CBOs believe should be under their own control	1. The inclusion of community advisory boards which helps to involve community members centrally has helped some IRBs understand the need to transcend traditional models for human subjects review 2. Preparation of extensive memos to university IRBs that lay out the history and practices of CBPR, bolstered by extensive in-person dialogue and email with IRB staff 3. Demonstrate to the IRB how other researchers at prominent institutions have successfully carried out collaborative work like CBPR while observing sound ethical practices, and that other IRBs have approved such multi-partner collaborative research. 4. Demonstrate to the IRB that community partners are experienced in scientific research and well-versed in human subjects protection protocols	1. Make sure academic IRBs know community partners 2. IRBs can keep well-informed of CBPR and other cutting-edge research approaches 3. IRBs can develop routine procedures for the review of CBPR projects 4. Provide clear guidance and tools for navigating IRB issues unique to academic-community collaboratives 5. Reassess how IRBs oversee situations in which participants desire access to and disclosure of their own study results 6. Work with community and tribal IRBs 7. Share positive models and problematic experiences with other teams, IRBs, and funding institutions 8. Research IRB members to assess their familiarity with CBPR and expertise in the field 9. Implement and invite IRB and human subjects administration staff to CBPR workshops to improve their overall understanding of the principles of CBPR10. Establish regular communication between researchers and IRBs
Hueston *et al*., 2006 [43]	To convene a panel to review issues of data integrity and participant protection in educational research, community-based participatory research, and research conducted by practice-based networks and summarize the issues and recommendations of this expert panel	1. Communities face the challenges of whether a community review and approval process are in place and the level of importance of the community’s decision	Not Available	1. There needs to be an ethical review of the CBPR project by both the IRB and the participating community 2. IRBs need to consider whether they have adequate knowledge of CBPR 3. IRBs need to consider whether appropriate community members are included in the process 4. IRBs need to consider how they can review an application in which the researcher proposes to develop the research focus and methodology with the community members 5. IRB oversight in cases of CBPR should include attention to the protection of the reputation of a community and the possibility of stigmatization of community members in addition to the protection of individual participants. 6. Investigators conducting CBPR should assure that all research projects are reviewed and approved by community representatives in addition to or as part of an IRB 7. For CBPR, the dissemination plan should be anticipated and reviewed, particularly for the dissemination of sensitive (i.e. potentially stigmatizing) results 8. Data from CBPR research should be made available for additional analyses only with the consent of the participating community and the original investigators
Malone *et al*., 2006 [17]	To examine the implications of the refusal of the university IRB to approve a CBPR study on cigarette sales practices in an inner-city community	1. IRB refused to approve modification because of the legality around buying and selling single cigarettes 2. IRB did not see community research partners, as researchers but rather as subjects 3. Community research partners felt betrayed by IRB’s rejection, feeling as though the IRB chose to protect community predators over the health of the community itself	1. Clarifying the relationship and duties and roles of research partners help the IRB process. 2. Having funders write a letter on behalf of the research will help the IRB process 3. Providing evidence-based research to back up claims also helps with IRB approval	1. Including a CBPR expert or ethicist in the IRB review of CBPR studies

CBPR, community based participatory research; CEnR, community-engaged research; CTSI, Clinical and Translational Science Institute; IRB, Institutional review board

### Challenges and Promising Practices for Engaging with IRBs

#### Challenge #1: Community partners not being recognized as equal research partners

One of the biggest challenges CEnR researchers reported was that IRBs often did not recognize community partners who are interested in engaging in human subjects research (HSR), as equal research partners/stakeholders in the process of CEnR research, resulting in delays or difficulties in receiving IRB approval in a timely manner. Some of the factors related to this challenge include the following: (1) Hesitancy reported by IRBs, when CEnR researchers include non-university affiliated clinics and staff as research partners; (2) Not having a standardized mechanism or process in place for non-affiliated community partners to serve as research partners within academic institutions; (3) Concerns related to the inability of the IRBs to ensure oversight of HSR protections for partnered community organizations who are interested in engaging in the process of research but are not affiliated with the academic IRB, which could lead to possible HSR violations.

Malone *et al.* [17] highlight an example of the IRB failing to the role of community partners as research partners. Researchers at the university hoped to use a CBPR approach where community partners would attempt to purchase single cigarettes to examine the impact of cigarette sales practices in an inner-city community in San Francisco, California. When researchers sought IRB approval, the IRB misunderstood the relationship between the community partners and the academic researchers and assumed researchers were using money to buy the help of community partners to commit the illegal act of buying single cigarettes. The IRB rejected this research proposal as they viewed it as a violation of HSR. This further hampered the research process as community partners felt betrayed by the IRB’s rejection, feeling as though the IRB chose to protect community predators (i.e. those selling cigarettes) over the health of the community itself [17]. This challenge highlights the need to not only clarify the roles, responsibilities, and relationships of research partners in the research process but also to include CBPR experts or ethicists in the IRB review of CBPR studies. Additionally, it showcases how IRBs are more inclined to protect institutional power at the expense of community partnership and collaboration [17,23].

### Recommendations and Lessons Learned

Some recommendations and lessons learned used by CEnR researchers to mitigate these challenges include allowing community partners to participate in certain aspects of the study if they completed additional training in CBPR and HSR from the institutions’ Clinical and Translational Science Institute (CTSI), in addition to the basic HSR training required for all participants [14]. Dissemination of HSR training that is accessible to all community investigators prior to IRB engagement was also noted as an important way to satisfy IRB concerns around HSR [14,22]. This demonstrates to the IRB that community partners are experienced in scientific and human subjects protection protocols [23]. Additionally, explicitly clarifying the academic-community partner relationship in the IRB protocol further supports the partnered process. This includes specifying the duties and roles of the community partners as well as the methods of community engagement with community research partners. Doing so provides the IRB with transparency and more insight into the academic-community collaborative relationship, which can help improve the IRB approval process [17]. Finally, academic investigators conducting CEnR should ensure that all research projects are reviewed and approved by community representatives in addition to or as part of an IRB process. Andrews and colleagues described utilizing several of these strategies to address the challenges associated with including community members as coinvestigators on the IRB protocol [22]. For example, the study sought to ensure an institutional faculty member with CBPR knowledge and experience served as part of the IRB. The program also provided training to IRB administrators, faculty, and community partners on the IRB process for CEnR. Additionally, the program implemented a process to allow community partners access to the electronic IRB application and acknowledgment as a coprincipal investigator, as well as instructional guidelines, templates, and algorithms to enhance the IRB navigation process [22].

#### Challenge #2: Cultural competence, the language of consent forms, and literacy level of partners

The second major challenge in CEnR research includes obtaining alternative forms of consent outside traditional methods from immigrant and vulnerable populations. Studies have noted the difficulty in framing and accommodating certain cultural nuances unique to specific populations participating in the research process in IRB protocols and consent forms (i.e. cultural nuances that may fall outside the boundaries of western values inherent to the concept of informed consent) [16]. This also includes ensuring consent forms match literacy levels for community partners in addition to IRB protocol standards. Some of the factors related to these challenges include: (1) Difficulty in obtaining consent forms from immigrant populations (due to concerns about immigration violations) as well as vulnerable populations like sex workers or unhoused individuals who may be wary of documentation [16,18,24]; (2) IRB-approved written consent form templates language that is too technical/complex to translate in a way that would properly capture and summarize the nature and content of the research study. These complexities can often prove difficult for non-English speaking communities with a strong oral language culture [18]; (3) IRBs preference for written documentation of consent. In some communities (e.g. undocumented immigrants, etc.), community members may be averse to signing any form of documentation due to fears of repercussions, even though they are willing to participate in research.

For these reasons, in some cases, verbal consent might be the only way these groups will agree to participate in the study.

### Recommendations and Lessons Learned

Some recommendations and lessons learned that researchers have used to mitigate these challenges include providing supervision for non-native-speaking study participants by engaging bilingual community leaders to help facilitate verbal consent and, in some cases, written consent. In addition, obtaining human studies-certified interpreters for non-native speakers would help in the consent process [18]. Convening an IRB with members educated in cultural nuances of the study population can help necessitate flexibility in the research project [16]. This can be especially beneficial for CBPR projects, which often need to have sufficient leeway for the project to adapt and tailor materials as needed to fit the community [18]. In addition, conducting studies involving refugee communities and other immigrant groups may necessitate educating IRBs on cultural nuances that may not conform to western values of individual autonomy, self-determination, and freedom inherent to the concept of informed consent. Finally, IRBs can provide a waiver of written/signed informed consent, wherein documentation of verbal consent obtained through an interpreter is sufficient for community members to participate in the research process [16]. One example of this is the Pirie et al study. The goal of the study was to assess and control occupational health risks among immigrants in Somerville, Massachusetts using bilingual teen educators to gather information on self-identified immigrant workers living or employed in the city. One of the major study challenges was that the existing IRB-approved written informed consent form was too technical/complex and written at too high of a literacy level for community members to understand and provide consent. The IRB approval process was further delayed because several corrections were required to improve the survey flow for participant comprehension. To address these challenges, the study included bilingual adult community leaders to facilitate participant verbal consent for immigrant community members who were non-native speakers. In addition, human studies-certified interpretation was also available for non-English speakers. One unexpected outcome from the delay in IRB approval was that it provided an opportunity for community members and research staff to deepen mutual relationships of trust, expand community capacity through education in human participants' protection, and support the Collaborative Institutional Training Initiative (CITI) certification of community partners. [18]

#### Challenge #3: IRBs apply formulaic approaches to CEnR

IRBs often review studies using a biomedical and behavioral framework, which may be inappropriate to the methodology, objectives, and purpose of CEnR [23]. CBPR, in particular, is characterized by community involvement as collaborators in the research process, reporting back results to study participants and a bidirectional relationship between the research team and community members. In some instances, these processes may be unfamiliar to IRB review boards. Some of the factors related to these challenges include: (1) The nature of the CBPR process often poses a challenge to the IRB’s determination of which party (i.e. research team versus community collaborators) is in control of the flow of data produced by the study (2) IRBs are concerned with how data should be shared with community participants (3) IRBs are concerned with the nature and length of the relationship with the community.

### Recommendations and Lessons Learned

Some recommendations and lessons learned that researchers have used to mitigate this challenge include conducting training for IRB reviewers to help them understand the principles of CEnR. Training can streamline the approval process by reducing delays and increasing the flexibility of the academic IRB review process. This can be implemented by inviting IRB and HSR administrative staff to CEnR-focused workshops to improve their overall understanding of the principles of CEnR, which in turn can help establish regular communication between researchers and IRBs [14,23]. In addition, including faculty members with CEnR knowledge and experience or CEnR experts/ethicists, in the IRB review of applicable CEnR studies, will help the review process, as they can serve to educate other IRB members on the CEnR principles and methodology during the research process [17]. These experts can also work with IRB offices to develop processes supportive of CEnR, and training tools for community investigators in order to remove any barriers in the IRB review process. Overall, increasing IRBs' understanding and knowledge of CEnR will lead to strengthening their community composition, including community-level ethical considerations for policies, processes, and application forms.

One example of this is the Chatterjee et al study, which was a pilot nutrition program launched by community partners and evaluated by investigators at Harvard Pilgrim Health Care Institute (HPHCI). The goal of the study was to evaluate the impact of a new school food program on the health of students. The study was designed as a community–academic partnership where school staff and academic health centers were involved as stakeholders in the research process. At the study’s outset, the IRB expressed concerns about several components of the CBPR study, including supervision for HSR protections at a remote site from the academic institution. Because of these rigid requirements with regard to HSR protocols, an imbalance of power was created by prioritizing the academic institution’s demands over the needs of the community organizations. To address these challenges, the study investigators utilized training materials developed by Harvard’s Clinical and Translational Science Center (CTSC) for community investigators in CBPR projects to train community members in the research process. The training covered important topics for all investigators to understand when conducting HSR and allowed community/student investigators to participate in certain aspects of the study if they completed training from the institution’s CTSC on HSR and CBPR [14]. Overall, working with IRB offices to develop processes supportive of CEnR and training tools for community investigators can remove many barriers and maintain long-term relationships in communities. Training IRBs to understand CEnR principles also streamlines, reduces delays, and increases the flexibility of the academic IRB review process [14].

#### Challenge #4: Extensive time duration for IRB preparation and approval has the potential to stifle the relationship with community partners

The final challenge highlighted from the scoping review includes extended durations for preparing and submitting CBPR/CEnR research, which prolongs the approval process. These extensive time periods for the IRB review can often stifle the research process and the relationship/partnership with community stakeholders as well as community research partners. This challenge is heightened for researchers working with multiple IRBs such as local, tribal, and national IRBs, in addition to academic IRBs. Some of the factors related to these challenges include (1) different types of IRBs often have varying interests and stakes in the research process; (2) IRBs can have different review requirements, and processes which can contribute to extended processing and approval time (e.g. local and tribal IRB policies) [15].

### Recommendations and Lessons Learned

Some recommendations and lessons learned from CEnR literature that researchers have used to help ease or speed up the review and approval process include identifying key stakeholders with influence in the community who can provide support with community buy-in for the research study [15]. Involving community partners in the research process and with the IRB as early as possible will also help with the review process, by minimizing any last-minute surprises that require revision or change in a study design or protocol [24]. Finally, implementing a process to allow community partners access to the electronic IRB application and acknowledgment as a coprincipal investigator, as well as instructional guidelines, templates, and algorithms to enhance the IRB navigation process will expedite the process. By doing so, community partners have a better understanding of the review process, which reduces the discordance between academic researchers and community partners in terms of the CEnR project.

One example of identifying key stakeholders can be found in the Holkup et al study. The goal of the study was to use family conference models to resolve problems with American Indian families struggling to care for an elder or resolve problems with a mistreated elder within a family system. This model provides a way for families to resolve problems while maintaining self-determination. The study encountered challenges concerning IRB requirements for informed consent. The structure approved by the IRB for gaining informed consent for the study disrupted the natural flow of conversations and the group dynamics of the group interviews. This study utilized action research process, which is a characteristic of CBPR that uses a cyclical and iterative process of planning, reflecting, reporting, and re-planning [26,27]. As a result, there was some difficulty in gaining true informed consent because of the nature of action research, to allow the project to evolve as the research progresses. It was difficult to specify explicitly what involvement in the research will mean for the participants [15]. To address these challenges, the study team identified key stakeholders with influence in the community who could provide support and improve buy-in within the community and the tribal IRBs. This sped up the process by reducing the scrutiny of the tribal IRBs to commence the research study.

In another example conducted by Hyatt et al, the goal of the study was to determine if an educational and training intervention involving both community partners and IRB leadership could resolve gaps in CBPR knowledge and mistrust. The study gave community leaders, particularly leaders in partner organizations, additional experience and training in survey development, implementation, and analysis, with assistance provided by co-investigators at Tufts University. The major challenge in working with immigrant workers was that protecting human subjects was a critical issue in which many community partners were not knowledgeable about the need for such protections or the role of the IRBs. To address and ease the review process, the lead investigators communicated IRB issues as often and as clearly as possible with both community partners and their own IRB to minimize last-minute delays and surprises. These communications were followed up with a written memo to both community partners and the IRB.

## Discussion

CEnR is characterized by direct community involvement/collaboration and equal partnership with community members in the research process [4]. Specifically, this bidirectional relationship takes into consideration multiple types of stakeholders in the research process and ensures that interventions are well-tailored and culturally and linguistically appropriate for the communities in which it will be implemented. This is often recognized as a key process for possibly reducing health disparities that result from structural racism [28]. CEnR provides an alternative approach to research compared to traditional forms of biomedical research which often separates interventions from its community context [15].

Increasingly, funding organizations, researchers, and communities recognize CEnR approach as an important methodology for understanding and addressing critical health concerns within communities [29,30]. This is because of the collaborative approach CEnR utilizes, which establishes community members as equal partners in the research process and recognizes the unique strengths each partner brings to the research [30].

IRBs ensure that research studies comply with applicable ethical standards and policies that protect research participants in a study. Our goal for this review was to identify barriers and hurdles that CEnR researchers encounter in seeking IRB approval for CEnR research and recommendations/lessons learned that circumvent these obstacles to obtaining IRB approval. Fifteen articles were included in this review and four categories of challenges were identified with subsequent lessons learned: (1) community partners not being recognized as research partners; (2) cultural competence, the language of consent forms, and literacy level of partners; (3) IRBs apply formulaic approaches to CEnR; and (4) extensive time duration for IRB preparation and approval has the potential to stifle the relationship with community partners.

Situating the findings from our scoping review into broader conceptual frameworks on community engagement may provide a roadmap for understanding how institutional and structural practices in IRBs can be enhanced to foster community engagement in research. For example, the Assessing Community Engagement (ACE) Conceptual Model demonstrates the dynamic relationship required to achieve health equity and systems transformation for health through meaningful community engagement [31]. Domains specific to ACE can serve as possible drivers of change to improve IRB practice of CEnR and promote health equity.

Table 4 below summarizes the challenges and strategies to mitigate these challenges and recommendations for improving the review process. We have also included domains from the ACE Model to articulate opportunities for improving IRB practice as it relates to CEnR.

**Table 4.** Summary of challenges and recommendations for CEnR researchers engaging with IRBs

Challenges	Strategies to mitigate challenges	Importance of recommendations	ACE Domain and opportunities for improving IRB practice
Community partners not being recognized as equal research partners [17,22] Issues with using nonuniversity affiliated clinics and clinic staff for recruitment No standardized process in place for community partners to as serve research partners with academic institutions IRB concerns around human subject violations for community organizations that are not under an affiliated IRB	– The IRB allowed community/student investigators to participate in certain aspects of the study if they completed training from the institution’s Clinical and Translational Science Institute (CTSI) on human subject research and CBPR in addition to the Human Subjects Research (HSR) training required- Disseminating human subjects research training that is accessible for all community investigators, including student investigators, will be an important way to satisfy IRB concerns – Clarifying the relationship and duties and roles of research partners helps the research review and approval process- Make sure academic IRBs know community partners – Provide clear guidance and tools for navigating IRB issues unique to academic-community collaboratives – Train community partners in human subject research to demonstrate to the IRB that community partners are experienced in scientific and human subject protection protocols- Investigators conducting CBPR should assure that all research projects are reviewed and approved by community representatives in addition to or as part of an IRB	– Leverages the opportunity for academic researchers to have direct access to contextualized local data otherwise not captured by traditional forms of biomedical research- Enhances the interpretation of research findings through an understanding of the local context provided by community partner involvement	*Strengthened Partnerships and Alliances*: qualities of the IRB that will foster and strengthen alliances with community partners include shared power between IRBs and community members during the review processes, where community partners can be involved in codesigning and developing the research partnership’s shared vision, goals, and responsibilities. In turn this, level of involvement and commitment by community partners can help build trust and promote sustained relations in which community partners are regarded as equalpartners.
Cultural competence, the language of consent forms, and literacy level of partners [16,18] Difficulty in obtaining consent from immigrant and vulnerable populations Consent form language and content are often too complex and difficult to understand Use of written consent versus verbal consent	– IRB provides a waiver of written/signed informed consent, wherein documentation of verbal consent obtained through an interpreter is sufficient – Supervision of non-native speakers by a bilingual adult leader helped to provide verbal consent- Human studies certified interpretation was available for non-English speakers – Having a review board that is sensitive to cultural concerns and therefore will allow necessary flexibility to the research project, especially for projects that have a CBPR component which often needs to have sufficient leeway for the project to follow its natural evolutionary course – Conducting studies involving refugee communities may necessitate educating IRBs on cultural nuances that may fall outside the boundaries of Western values of individual autonomy, self-determination, and freedom inherent to the concept of informed consent	– Allows for the participation of vulnerable populations who often are inaccessible or understudied e.g. undocumented individuals, sex workers, unhoused individuals, etc.	*Expanded Knowledge*: co-creation of new insights ideas and resources and tools can facilitate bi-directional learning between academic institutions and communities engaged in research, which in turn can improve IRBs' cultural competencies and understanding cultural nuances related to engaging specific populations in community research
IRBs apply formulaic approaches to CEnR [14,23] IRBs are formulaic in their approach to the review process The review process is often designed around biomedical and behavioral research	– Training IRB’s to understand CEnR principles – Strategies such as working with IRB offices to develop processes supportive of CEnR and training tools for community investigators can remove many barriers – Community advisory boards allow for greater community member involvement – Implement and invite IRB and human subject administration staff to CEnR workshops to improve their overall understanding of the principles of CEnR and to establish regular communication between researchers and IRBs – Including a CEnR expert or ethicist in the IRB review of CEnR studies – Having an institutional faculty member with CEnR knowledge and experience to be part of the IRB group – Encourage IRBs to increase their understanding of CEnR strengthen their community composition, and explicitly include community-level ethical considerations, in their policies, processes, and application forms	– Streamline the IRB review process by reducing delays and increasing the flexibility of the IRB review process, and IRB members will understand the intricacies associated with CEnR – Community advisory board members make up members of the community whose insights can help to inform and modify research approval methods to better fit the CEnR framework	*Expanded Knowledge*: co-creation of new insights ideas, resources, and tools focused on community engagement and community partnerships can facilitate bi-directional learning between academic institutions and communities engaged with CEnR
Extensive time duration for IRB preparation and approval has the potential to stifle the relationship with community partners. [15,24] Working with multiple IRBs (local, tribal, and national IRBs in addition to academic IRBs) Hurdles around local and tribal policies to obtain IRB approval	– Identifying key stakeholders with influence in the community who can provide support for the research study – It is critical for academic researchers to involve their community partners with the IRB as early in the research process as possible – Implement a process to allow community partners access to the electronic IRB application and acknowledgment as a coprincipal investigator, as well as instructional guidelines, templates, and algorithms to enhance the IRB navigation process	– Reduce delays in the review process – Maintain community engagement in research	*Improved Health and Health Care Programs and Policies*: designing community-aligned solutions that are sustainable and actionable, can help maintain community-engaged partnerships in the research process.

ACE, assessing community engagement; CEnR, community-engaged research; CTSI, Clinical and Translational Science Institute; HSR, human subjects research; IRB, Institutional review board.

Overall, the challenges highlighted in this review demonstrate the gaps that exist in obtaining approval for CEnR research. The concern of IRBs failing to recognize community partners as equal partners in the research study stems from the inability of the IRB to have true oversight of community partners involved in research, especially when community partners are not affiliated with academic IRBs. While this might be a legitimate concern, the nature of CEnR requires community partner involvement in the research process as key contributing stakeholders. By allowing community partners to participate in the research process, it leverages the opportunity for academic researchers to have direct access to contextualized local data otherwise not captured by traditional forms of biomedical research.

Secondly, it enhances the interpretation of research findings through an understanding of the local context provided by community partner involvement and provides opportunities for building human capital and a community resource infrastructure that can directly affect changes in local policy through participating community partners [32]. As such, IRBs need to recognize that the involvement of the community and its members as research partners as opposed to research subjects, maximizes community benefits and minimizes harm, which can lead to improved public health [30].

From our findings, to ease IRB concerns about potential human subject violations, training community partners or involving them in workshops that cover HSR training (e.g. CITI training) can ease IRB concerns and help them perceive community partners as trained stakeholders with a basic understanding of HSR.

IRB review processes are rooted in biomedical and behavioral research frameworks and often do not have the flexibility needed to review CEnR studies [9,23]. Therefore, it is imperative for institutional IRBs to be trained in the principles of CEnR and CBPR methods. This can assist in reducing delays associated with the review process as IRB members will understand the intricacies associated with this type of research [14,23]. Some ways to address this can include: (1) having IRB members participate in CBPR and CEnR workshops to gain a better understanding of the process; (2) creating a knowledge exchange program between community advisory boards and IRB members; and (3) including institutional faculty members with CBPR or CEnR experience as members of the IRB. All of these activities can provide IRBs with increased knowledge and exposure to CEnR; thus, removing barriers to the ethical review process and shortening the extensive time duration for IRB review and approval.

CEnR engages different community groups, which means that other IRBs like tribal or local IRBs may have to be involved in the ethics review process. Identifying key stakeholders with influence in the community to promote buy-in of the CEnR research project can help to speed up the research process. Through the influence of key stakeholders, community members are much less hesitant to participate and are more accepting and willing to engage in the research process. In addition, engaging and involving community partners early on in the research process can also help reduce time constraints that may arise during the review process [15,24].

More so, IRBs must understand that certain accommodations that are nontraditional to biomedical research may need to be included for CEnR or CBPR studies to account for language barriers and varying literary levels among community partners and allow for greater cultural competency. The inclusion of bilingual interpreters to provide verbal consent for partners, or allowing verbal consent without having partners sign a document will be key for involving some vulnerable groups like undocumented immigrants, etc. Summarily, IRBs need to be flexible in the research process, especially for studies involving vulnerable communities whose cultural context may differ from western norms and values [16,18].

Our aggregated findings are consistent with individual reports from CEnR investigators. For example, an article on overcoming barriers to effective CBPR research in US medical schools reported institutional barriers to their CBPR associated with limited understanding of CBPR, the perception that CBPR lacks rigor, and concerns of objectification of the community in research [33]. Another study reported results of a content analysis of 30 institutions and found that review boards often favored traditional biomedical research frameworks, which could unknowingly set this as the standard for all forms of research [34]. Similar to our review other studies recommend that IRBs receive training in the principles of CBPR and IRBs should require CBPR investigators to document the process of key decision-making as regards the study design and community consultation related to the design [34,35].

There are several strengths to this study. First, CEnR researchers have encountered and discussed some barriers to IRB approval [21,24,36]. This is the first scoping review that documents the challenges that CBPR investigators have with obtaining ethical approval from IRBs. Our review generated a total of 15 articles published over the course of 12 years, increasing in frequency in the 2010s and spread across numerous journal types. The diversity in journal types and disciplines that have explored this topic as well as the increased frequency of publications on this topic over the last two decades points towards the growing relevance and prominence of CEnR across disciplines and highlights the role this scoping review can play in coalescing a knowledge base on IRB practice.

Additionally, this review reports on lessons learned from the field and recommendations that can assist CBPR investigators in obtaining approval from ethical review boards. The collated recommendations from this study provide a catalog of strategies that IRBs can adopt to understand CBPR methodologies and be inclusive of community partners in the research process, which enriches the research process and contributes to capacity building for community partners who may seek to conduct their own future research. This review is also timely as it complements the growing requests from funders (e.g. NIH) for more collaborative and community-engaged approaches to tackling health disparities, structural racism, racism in healthcare, community mistrust, and health inequities [8,37–40].

There are several limitations to this study. This scoping review included a combination of research studies and expert opinions/reports. While these expert reports are important, they include second-hand accounts of the researchers’ experiences seeking ethical approval for CBPR and are not primary research, which might have provided stronger evidence for this topic. Another limitation of the review was that many studies included were not consistent in reporting demographic data of the community partners or target population, which made it difficult to examine and report on any relationship between the target population and some of the IRB approval outcomes of the study. Only a few studies reported on the process of gaining approval from the IRB, and only one study reported on the process of denial from the IRB. Most of the studies that we reviewed reported obtaining IRB approval, but had limited to no details on their experience in engaging the IRBs, nor did they include a related process paper on their engagement with the IRB. Regardless of approval/disapproval, more studies might consider documenting their process of engagement with IRBs both academically and within the community. In addition, many of the recommendations reported in the review are specific to the nature and context of the research topic and might not be generalizable to all forms of CEnR. Several studies reported on potential recommendations that might ease the process, however, Investigators had not tested or implemented these recommendations in their study settings; therefore, there was no evidence or outcomes on the success of these recommendations. It is also important to note that some of the challenges encountered in CEnR studies also exist in some traditional forms of biomedical research studies (e.g. the need for consent to be understandable and available in languages spoken by the community members). Future studies can implement these recommendations to document if and how they might work across different community study settings and with different community partners.

Our findings are important given the 2021 statement from the Association for the Accreditation of Human Research Protection Programs (AAHRPP) for the support of community engagement as a result of the pandemic [41]. The standard put forth by AAHRPP has always included a requirement to engage in community research, the urgency for this requirement was further highlighted by the COVID-19 pandemic, the consequences of which disproportionately affected the most vulnerable communities in the USA.

The standards state that AAHRPP-accredited organizations remain committed to finding innovative, effective ways to engage and protect vulnerable communities. The standards include: (1) Following written policies and procedures that establish a safe, confidential, and reliable channel for current, prospective, or past research participants or their representatives that permits them to discuss problems, concerns, and questions; obtain information; or offer input with an informed individual who is unaffiliated with the specific research protocol; (2) Conducting activities designed to enhance the understanding of human research by participants, prospective participants, or their communities, when appropriate; (3) Promoting the involvement of community members, when appropriate, in the design and implementation of research and the dissemination of results [41].

This is an important statement from the AAHRPP, as it means there is less of a need to advocate for the inclusion of community engagement in the IRB process, and more need to help IRBs and researchers learn effective strategies for their settings and context.

In conclusion, findings from our study suggest that IRBs might benefit from more training in CEnR, its requirements, and its methodologies. Future studies should seek to engage, maintain regular communication with IRBs and educate IRBs on the CEnR research process, identify lessons learned to advance the field, and consider flexible and alternative processes for ethical review and approval of CEnR compared to traditional biomedical research studies, which view community members as research subjects instead of recognizing community members as research partners.

## Supplementary material

For supplementary material accompanying this paper visit https://doi.org/10.1017/cts.2023.516. click here to view supplementary material
