# Supplementary material for: Challenges and recommendations to improve institutional review boards’ review of community-engaged research proposals: A scoping review
Source: J Clin Transl Sci. 2023 Mar 31;7(1):e93. doi: 10.1017/cts.2023.516 (PMC10130837; doi:10.1017/cts.2023.516)
Supplement: Supplementary file 1 [file S2059866123005162sup001.docx]

Appendix – MEDLINE search

|  | **Searches** |
| --- | --- |
| 1 | Community-Based Participatory Research.mp. or exp Community-Based Participatory Research/ |
| 2 | (community consent or community risk).ti,ab. |
| 3 | (communit* adj3 (particip* or involv* or engag* or collabor* or base*) adj3 research).ti,ab. |
| 4 | institutional review board.mp. or exp Ethics Committees, Research/ |
| 5 | irb.ti,ab. |
| 6 | informed consent.mp. or exp Informed Consent/ |
| 7 | exp Ethics, Research/ |
| 8 | (ethic* adj3 (research* or committee* or review* or train*)).ti,ab. |
| 9 | 1 or 2 or 3 |
| 10 | 4 or 5 or 6 or 7 or 8 |
| 11 | 9 and 10 |
